# Tumor size predicts prognosis of head and neck synovial cell sarcoma

**DOI:** 10.3892/ol.2014.2634

**Published:** 2014-10-24

**Authors:** ALIMUJIANG WUSHOU, XIN-CHAO MIAO

**Affiliations:** 1 Cancer Research Institute, Fudan University Shanghai Cancer Center; Department of Oncology, Shanghai Medical College, Fudan University, Shanghai 200032, P.R. China; 2 Department of Oral and Maxillofacial Surgery, Ninth People’s Hospital, Shanghai Jiao Tong University School of Medicine, Shanghai 200011, P.R. China

**Keywords:** synoviosarcoma, head and neck, treatment, outcome, meta-analysis

## Abstract

Head and neck synoviosarcoma (HNSS) is uncommon. To the best of our knowledge, the specific clinicopathological characteristics, treatment outcome and prognostic factors of HNSS were uninvestigated at the time of writing, so a meta-analysis was performed. An online data collection was carried out using PubMed and Google Scholar. Studies that reported primary HNSS and the treatment, follow-up time and outcome were chosen for the present study. In total, 93 cases from 26 studies were included for analysis. The study sample consisted of 55 males and 38 females and the median age was 32.1 years (range, 4–76 years). The median follow-up period was 62.1 months (range, 1–373 months). The tumor size was correlated with local recurrence and metastasis of HNSS, as well as with mortality (P=0.001, P<0.0001 and P<0.0001, respectively). The three-year, five-year and 10-year survival rates were 82.1, 80.4 and 78.2% for treatment with surgery alone, and 88.5, 85.5 and 82% for treatment with surgery plus radiotherapy, respectively. A significant tumor size-dependent difference was found between the overall survival (OS) rates (P<0.0001), as tumors that were >5.0 cm in diameter were associated with a worse OS rate (hazard ratio, 6.460; 95% confidence interval, 206–18.917; P=0.001). The tumor size was found to be an independent adverse prognostic factor for the OS of HNSS patients. In conclusion, surgical excision is a mainstream treatment of HNSS and post-operative adjuvant radiotherapy improves the OS rate of HNSS patients.

## Introduction

Synovial cell sarcoma, or synoviosarcoma, (SS) is a mesenchymal malignancy that is termed SS since its histological appearance is similar to that of the synovium. However, SS rarely exhibits a synovial structure and is considered to originate from pluripotent mesenchymal cells (1). The characteristic biphasic pattern of SS is due to the two morphologically distinct but histogenetically related cell types that compose the sarcoma. Depending on the relative prominence of the two cell populations and the degree of differentiation, these tumors form a continuous histopathological spectrum of biphasic, monophasic fibrous, monophasic epithelial and poorly differentiated (round-cell) types (2). Since SS can be slow-growing, appear to be benign on imaging studies, vary in size and cause pain resembling that associated with trauma, SS is the most commonly misdiagnosed soft tissue malignancy (3,4). The diagnosis of SS is made on the basis of its relatively distinctive, yet markedly variable, histopathological appearance in conjunction with histochemical findings, immunohistochemistry, electron microscopy and cytogenetic analysis, which have proved valuable in confirming morphological diagnoses (5,6).

SS is a distinct soft tissue sarcoma that tends to be located in the extremities (2). The lower extremities account for ~70% of cases, whereas SS is uncommon in the head and neck region, with only 3% of SS tumors located there (7). Due to low clinical morbidity, non-specific symptoms and heterogeneous histopathological features, head and neck SS (HNSS) is often misdiagnosed (8). As a result, clinical diagnosis and treatment planning remain a challenge (9). To the best of our knowledge, there have been no controlled studies to define the optimal management protocol for HNSS, and the treatment methods reported include surgery, chemotherapy, radiotherapy and multiple treatment modalities, with variable results. In addition, no specific prognostic factors of HNSS have been reported to date. The aims of the present study were to review the clinicopathological characteristics of HNSS in head and neck patients, report and compare the treatment options, and identify the prognostic factors of mortality.

## Materials and methods

### Selection of studies

A systematic literature search was performed using PubMed and Google Scholar. The search strategy was based on the combination of text words: ‘Synoviosarcoma OR synovial sarcoma OR synovial cell sarcoma’, ‘head and neck region’, ‘upper aerodigestive tract’, ‘oral and maxillofacial region’, ‘sinonasal region’ and ‘neck’. For the literature search in PubMed, no lower date limit was utilized and the upper date limit was October 31, 2013. Despite the fact that no language restrictions were initially imposed, the full-text review and the final analysis were limited to studies published in English. The references of all the retrieved studies were searched for additional relevant studies to enlarge the scope of the literature search.

### Eligible criteria

A study was included for analysis if it reported a human study and histologically confirmed primary HNSS, provided a clear description of any treatment, reported a definite follow-up time of more than month, and provided the treatment outcome. The study was excluded if it reported recurrent or metastatic HNSS, or synchronous or metachronous multiple cancers in other organs or diseases, and if the study was a case series providing a mean or medium follow-up time.

### Data extraction

A data extraction sheet was developed. The data extracted for each patient consisted of the age, gender, tumor history, tumor presentation, tumor size, tumor extension, lymphadenopathy status, surgery type, surgical margins, presence of neck dissection, histological grade, adjuvant therapy provided, follow-up time and treatment outcome. Not all studies contained all these pieces of data; however, they were included in the present analysis if the treatment and outcome were provided. In certain cases, the patients had more than one treatment and, thus, only the final treatment received was included in the comparison of treatments.

### Statistical analysis

The χ^2^ or Fisher’s exact tests for categorical variables were used for two-group comparisons of the clinicopathological parameters. Differences in the numerical variables were assessed using Student’s t-test or non-parametric Wilcoxon test. Significant variables identified by univariate analysis were then entered into binary logistic regression models to identify independent predictors of mortality. The odds ratio and 95% confidence interval (CI) were reported for the logistic regression model. For time-to-event analysis, Kaplan-Meier curves were plotted and the log-rank test was used. Analysis of the effect of prognostic factors on cause-specific survival was undertaken using Cox proportional-hazards regression. When P<0.05, the difference was regarded as statistically significant. All the statistical tests were two-tailed and all the data were analyzed using SPSS 18.0 software for Windows (SPSS, Inc., Chicago, IL, USA).

## Results

### Patient demographics

**Figure 1:**
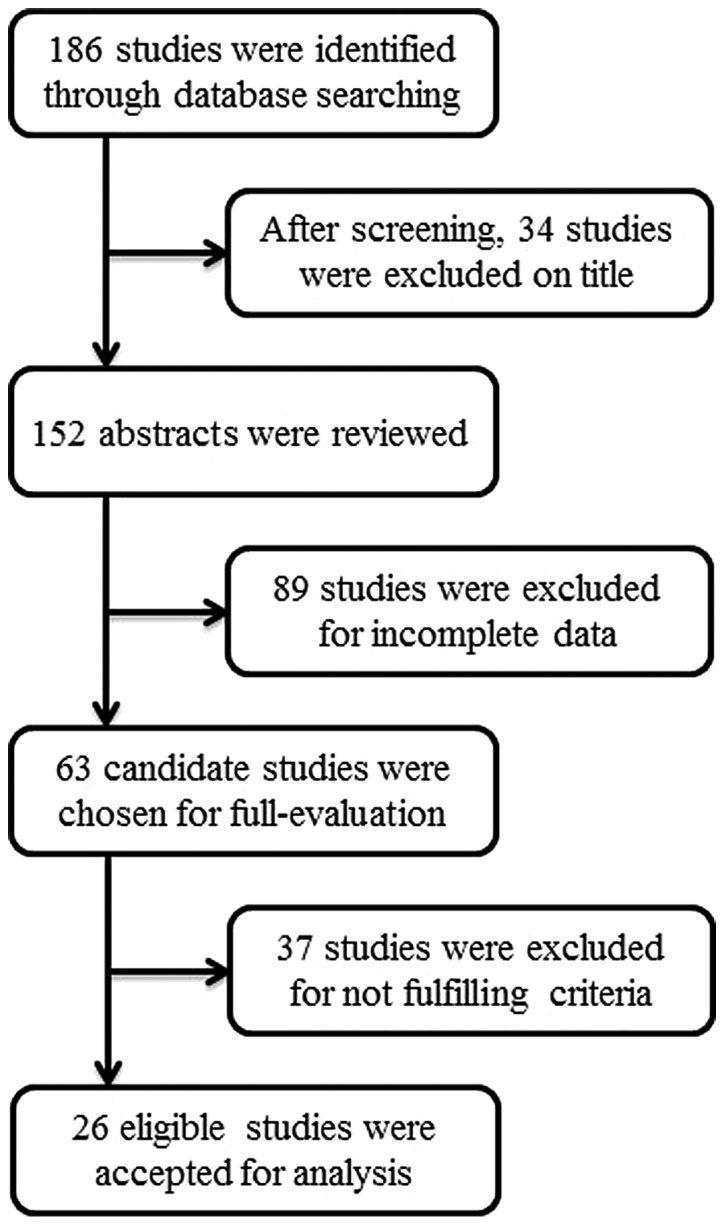
Flow diagram of the process of identifying and selecting studies for the analysis.

In total, 93 cases from 26 studies met the eligibility criteria for inclusion in the present analysis (8,10–34). The details of the identification and selection of the studies are presented in Fig. 1. The 93 patients consisted of 55 male and 38 female patients, providing a male-to-female ratio of 1.44:1.

The median age at the time of diagnosis was 32.1 years (range, 4–76 years).

### Tumor location, treatment and follow-up

In total, 50.5% of the tumors were located in the upper aerodigestive tract, 26.9% in the neck and 14.0% in the skull base. The treatment modalities consisted of surgery (41.9%), surgery plus radiotherapy (28.0%), surgery plus radiochemotherapy (20.4%) and other treatments (9.7%), including surgery with chemotherapy followed by radiotherapy. The median follow-up period was 62.1 months (range, 1–373 months). The baseline characteristics of the 93 HNSS patients are illustrated in Table I.

### Differential analysis between clinicopathological characteristics and outcome statuses

In order to identify the differences between the clinicopathological features of HNSS patients with different outcome statuses, the data of the 93 cases were categorized into three outcome groups, local recurrence, distant metastasis and survival. Each category was further divided into two groups, which resulted in the recurrence, recurrence-free, metastasis, metastasis-free, non-survival and survival groups (Table II). Significant differences in tumor size were identified between the recurrence-free and recurrence, metastasis-free and metastasis, and survival and non-survival groups (P=0.001, P<0.001 and P<0.001, respectively). In addition, significant differences were found in the pathological differentiation between the recurrence-free and the recurrence (P=0.008) and survival and non-survival groups (P=0.026). The logistic regression model was performed to evaluate the risk of recurrence, metastasis and mortality. The risk of tumor recurrence, metastasis and mortality was higher in the patients with a tumor >5.0 cm in diameter compared with those with a tumor ≤5.0 cm in diameter (Table III).

### Survival and Cox-regression analysis

**Figure 2:**
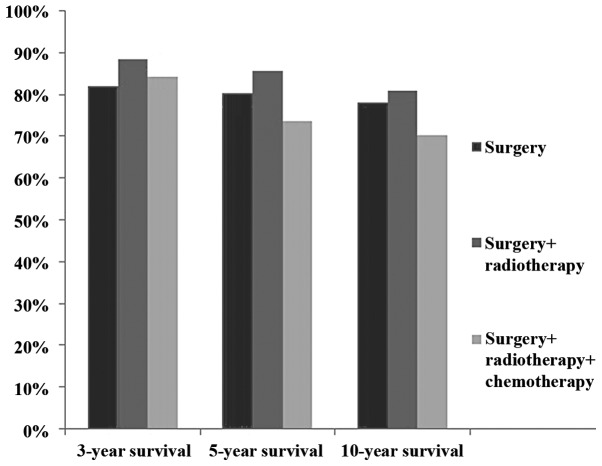
Overall survival rates of head and neck synoviosarcoma patients treated with three different treatment modalities.

**Figure 3:**
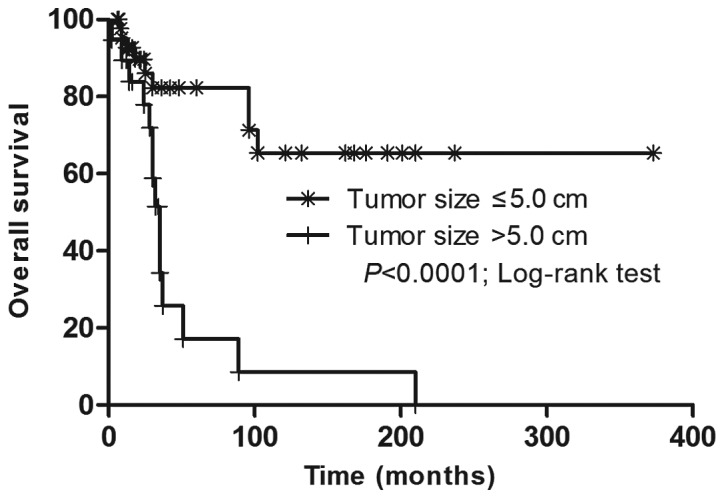
Kaplan-Meier analysis for overall survival of head and neck synoviosarcoma patients depending on tumor size.

In total, 20 cases relapsed following the first treatment and the recurrence rate was 21.5%. The distant metastasis and mortality rates were 18.3 and 26.9%, respectively. The three-year survival rate was 82.1% for surgery alone, 88.5% for surgery plus radiotherapy and 84.2% for surgery plus radiochemotherapy. The five-year survival rate was 80.4% for surgery alone, 85.5% for surgery plus radiotherapy and 73.7% for surgery plus radiochemotherapy (Fig. 2). Marked tumor size-dependent differences in the overall survival (OS) rate were revealed (Fig. 3). The Cox proportional-hazards model was utilized to predict the independent prognostic factors for OS. A tumor >5.0 cm in diameter was associated with a worse OS rate and the mortality risk increased by 6.460-fold (95% CI, 2.206–18.917).

## Discussion

To better elucidate whether the clinicopathological characteristics and treatment were correlated with survival in patients with HNSS and to find specific prognostic factors, a large meta-analysis of 93 patients with histologically confirmed primary HNSS was performed. Surgery is the major treatment for HNSS, resulting in a good prognosis, while surgery-based combined treatment modalities are not statistically superior to surgery alone. In addition, the patients with tumors >5.0 cm in diameter have a higher risk of local tumor recurrence, distant metastasis and mortality than those with tumors ≤5.0 cm in diameter. Importantly, the tumor size was the only independent adverse prognostic factor for determining the OS.

Approximately half of the tumors in the 93 cases were located in the upper aerodigestive tract. The upper aerodigestive tract and neck are the most common originating sites of HNSS and they account for 75% of HNSS. The tumor site determines the clinical presentation of HNSS. Clinically, HNSS is a painless and slow-growing mass, and is usually asymptomatic until it attains a size sufficient to create pressure on the adjacent structures. As a result, those in concealed locations, such as the infratemporal fossa and skull base, which are inaccessible for the clinical examination of a tumor in the early stages, grow unnoticed for a considerable period and the tumors are commonly found at an advanced stage.

Surgical excision is the mainstay of treatment for HNSS, according to the present study. In total, 93% of the cases were treated with surgery or surgery plus adjuvant therapy and resulted in a three-year OS rate of 85.3%, five-year OS rate of 81.4% and 10-year OS rate of 78.3%. The results in the present study were higher than those previously reported in the former largest analysis with 40 consecutive cases, by the University of Texas MD Anderson Cancer Center (Houston, TX, USA) (9). An explanation for this survival gap is that 19 of 40 cases possessed recurrent disease with positive surgical margins, and a robust association between negative margins and local recurrence-free survival was observed.

The present meta-analysis results are influenced by literature selection biases. However, existing data support the role of adjuvant radiotherapy in improving the local control of HNSS. The patients who received surgery plus radiotherapy achieved good local control and higher survival rates than those treated with surgery alone, although the difference was not statistically significant (P=0.19). The group of patients who underwent surgery plus radiochemotherapy possessed decreased five- and 10-year OS rates compared with the other two treatment modalities, although it is too soon to conclude that chemotherapy does not improve the OS rate of HNSS since six of the 19 patients in the surgery plus chemoradiotherapy group were diagnosed with advanced-stage disease, either with an extremely large tumor size with extension to adjacent structures, or the patients possessed multiple distant metastasis already. It may be concluded that the early detection of HNSS and total extirpation of the tumor, achieving negative margins, is more effective than employing a salvaging approach at a late stage of tumor development.

Another major interest of the present study was to identify the prognostic factors for HNSS patients. Prognosis in SS has been correlated with the patient age, tumor site, tumor size, mitotic rate, presence of necrosis and histological subtype (35–39). The present study confirms that the tumor size is the only unfavorable prognostic factor for HNSS survival. Certain early studies reported a more favorable outcome in patients with biphasic tumors, whereas other groups found no differences in survival between patients with monophasic tumors and those with biphasic tumors (36,39,40). The present results confirmed the lack of prognostic importance of the histological subtype, even though there were significant differences between the histological subtype and different outcome statuses (Table II).

A few limitations of the present study must be considered. Firstly, even though all the analyzed cases included the treatment outcome and follow-up, certain pieces of important information, including the pathological subtype, surgical margins and tumor extension, were not clearly specified in several cases. Missing these important clinicopathological parameters may influence the results of the present study. Secondly, it is extremely difficult to assemble single center or multicenter prospective trials for an uncommon disease such as HNSS. Thus, the retrospective data makes selection bias a possibility.

Despite its limitations, the present meta-analysis comprehensively analyzed the clinicopathological features of HNSS from the sporadic case reports in the peer-reviewed English literature to date. Surgical excision is a mainstream treatment of HNSS. Post-operative adjuvant radiotherapy is effective in local tumor control and improves the OS rate of HNSS. However, the effectiveness remains to be validated in further multicenter, longitudinal, prospective, large cohort studies. In addition, the present study confirmed that a tumor size >5.0 cm in diameter was an independent adverse prognostic factor for OS.

## Figures and Tables

**Table I:** Baseline characteristics, tumor site distribution and treatment type of 93 patients with head and neck synoviosarcoma.

Feature	Value
Age, years	
Median	32.1
Range	4–76
Gender, n	
Male	55
Female	38
Site, n	
Upper aerodigestive tract	47
Neck	25
Skull base	13
Other	8
Treatment type, n	
S	39
S+R	26
S+R+C	19
S+C+R	3
Other	6

S, surgery; R, radiotherapy; C, chemotherapy.

**Table II:** Clinicopathological differences in head and neck synoviosarcoma between different outcome statuses.

	Recurrence, n (%)				Metastasis, n (%)				Survival, n (%)			
									
Characteristic	No	Yes	Total	P-value	Yes	No	Total	P-value	Yes	No	Total	P-value
Age, years				0.165				0.256				0.907
≤32	27 (65.9)	14 (34.1)	41		32 (72.7)	12 (27.3)	44		39 (73.6)	14 (26.4)	53	
>32	26 (81.3)	6 (18.8)	32		26 (83.9)	5 (16.1)	31		29 (72.5)	11 (27.5)	40	
Gender				0.186				0.998				0.919
Male	28 (66.7)	14 (33.3)	42		34 (77.3)	10 (22.7)	44		40 (72.7)	15 (27.3)	55	
Female	25 (80.6)	6 (19.4)	31		24 (77.4)	7 (22.6)	31		28 (73.7)	10 (26.3)	38	
Tumor location				0.443				0.537				0.532
Superficial	17 (77.3)	5 (22.7)	22		18 (85.7)	3 (14.3)	21		20 (74.1)	7 (25.9)	27	
Moderate	10 (83.3)	2 (16.7)	12		10 (71.4)	4 (28.6)	14		12 (63.2)	7 (36.8)	19	
Deep	26 (66.7)	13 (33.3)	39		30 (75.0)	10 (25.0)	40		36 (76.6)	11 (23.4)	47	
Tumor size, cm				0.001				<0.001				<0.001
≤5.0	28 (77.8)	8 (22.2)	36		29 (82.9)	6 (17.1)	35		35 (79.5)	9 (20.5)	44	
>5.0	5 (29.4)	12 (70.6)	17		4 (26.7)	11 (73.3)	15		5 (26.3)	14 (73.7)	19	
Tumor extension				1.000				0.516				1.000
No	4 (100.0)	0 (0.0)	4		3 (100.0)	0 (0.0)	3		4 (100.0)	0 (0.0)	4	
Yes	12 (80.0)	3 (20.0)	15		8 (66.7)	4 (33.3)	12		13 (86.7)	2 (13.3)	15	
Surgical margins				0.228				1.000				1.000
Negative	14 (93.3)	1 (6.7)	15		11 (84.6)	2 (15.4)	13		14 (93.3)	1 (6.7)	15	
Positive	1 (50.0)	1 (50.0)	2		1 (100.0)	0 (0.0)	1		2 (100.0)	0 (0.0)	2	
Neck dissection				0.315				0.101				0.553
No	22 (84.6)	4 (15.4)	26		22 (88.0)	3 (12.0)	25		25 (92.6)	2 (7.4)	27	
Yes	5 (62.5)	3 (37.5)	8		4 (57.1)	3 (42.9)	7		7 (87.5)	1 (12.5)	8	
Histology				0.008				4.190				0.026
Monophasic	26 (76.5)	8 (23.5)	34		17 (77.3)	5 (22.7)	22		19 (65.5)	10 (34.5)	29	
Biphasic	22 (84.6)	4 (15.4)	26		22 (71.0)	9 (29.0)	31		28 (66.7)	14 (33.3)	42	
Unclassified	5 (38.5)	8 (61.5)	13		19 (86.4)	3 (13.6)	22		21 (95.5)	1 (4.5)	22	
Treatment type				0.828				0.116				0.803
Surgery	22 (75.9)	7 (24.1)	29		24 (88.9)	3 (11.1)	27		29 (74.4)	10 (25.6)	39	
Surgery + radiotherapy	20 (80.0)	5 (20.0)	25		33 (73.3)	12 (26.7)	45		21 (80.8)	5 (19.2)	26	
Surgery + radiochemotherapy	10 (71.4)	4 (28.6)	14		57 (79.2)	15 (20.8)	72		14 (73.7)	5 (26.3)	19	

**Table III:** Logistic regression analysis of risk factors for head and neck synoviosarcoma.

Characteristic	Odds ratio (95% CI)	P-value
Recurrence		
Tumor size >5.0 cm	8.400 (2.275–31.009)	0.001
Metastasis		
Tumor size >5.0 cm	13.292 (3.140–56.270)	<0.001
Mortality		
Tumor size >5.0 cm	10.889 (3.099–38.261)	<0.001

CI, confidence interval.
